# PKPD Modeling of the Inoculum Effect of *Acinetobacter baumannii* on Polymyxin B *in vivo*

**DOI:** 10.3389/fphar.2022.842921

**Published:** 2022-03-16

**Authors:** Alexia Chauzy, Grace Akrong, Vincent Aranzana-Climent, Jérémy Moreau, Laure Prouvensier, Hélène Mirfendereski, Julien M Buyck, William Couet, Sandrine Marchand

**Affiliations:** ^1^ INSERM U1070, Poitiers, France; ^2^ UFR Médecine-Pharmacie, Université de Poitiers, Poitiers, France; ^3^ Département de Toxicologie et de Pharmacocinétique, CHU de Poitiers, Poitiers, France

**Keywords:** inoculum effect, polymyxin B (PMB), *in vivo*, PKPD, modelling, *Acinetobacter* baumannii

## Abstract

The reduction in antimicrobial activity at high bacterial counts is a microbiological phenomenon known as the inoculum effect (IE). In a previous *in vitro* study, a significant IE was observed for polymyxin B (PMB) against a clinical isolate of *Acinetobacter baumannii*, and well described by a new pharmacokinetic-pharmacodynamic model. Few *in vivo* studies have investigated the impact of inoculum size on survival or antibiotic efficacy. Therefore, our objective was to confirm the influence of inoculum size of this *A. baumannii* clinical isolate on PMB *in vivo* effect over time. Pharmacokinetics and pharmacodynamics of PMB after a single subcutaneous administration (1, 15 and 40 mg/kg) were studied in a neutropenic murine thigh infection model. The impact of *A. baumannii* inoculum size (10^5^, 10^6^ and 10^7^ CFU/thigh) on PMB efficacy was also evaluated. *In vivo* PMB PK was well described by a two-compartment model including saturable absorption from the subcutaneous injection site and linear elimination. The previous *in vitro* PD model was modified to adequately describe the decrease of PMB efficacy with increased inoculum size in infected mice. The IE was modeled as a decrease of 32% in the *in vivo* PMB bactericidal effect when the starting inoculum increases from 10^5^ to 10^7^ CFU/thigh. Although not as important as previously characterized *in vitro* an IE was confirmed *in vivo.*

## Introduction

*Acinetobacter baumannii* is an opportunistic Gram-negative pathogen responsible for severe clinical infections encountered in intensive care units (ICUs) worldwide, such as acquired pneumonia and bacteremia but also urinary tract infections, meningitis and infections of traumatic wounds (García-Garmendia et al., 2001; Garnacho et al., 2003; Gil-Perotin et al., 2012; El-Saed et al., 2013; Garnacho-Montero and Timsit, 2019). Carbapenems are used as the first-line treatments for *A. baumannii* infections (Wong et al., 2017). However, due to the increase of *A. baumannii* strains resistant to carbapenems, other antibiotics such as polymyxins [colistin (CST) and polymyxin B (PMB)] may be used as last-line treatments (Wong et al., 2017; Garnacho-Montero and Timsit, 2019).

High bacterial counts may alter antibiotherapy success due to an inoculum effect (IE) (Chastre and Fagon, 2002; Koenig and Truwit, 2006; Kalanuria et al., 2014), corresponding to a reduction of the antibiotic activity as bacterial count increases (Harada et al., 2014; Lenhard and Bulman, 2019). This phenomenon has been described *in vitro* for ß-lactam antibiotics used against ß-lactamases-producing bacteria such as *Escherichia coli* and *Klebsiella pneumoniae* (Bedenić et al., 2001; Harada et al., 2014; Smith and Kirby, 2018; Lenhard and Bulman, 2019). It has also been reported *in vitro* with other antibiotics and bacteria, including glycopeptides used against *Staphylococcus aureus* (Rio-Marques et al., 2014), fluoroquinolones against *S. aureus* and *Pseudomonas aeruginosa* (Mizunaga et al., 2005), aminoglycosides against *S. aureus* and *E. coli* (Li and Ma, 1998) or PMB against *P. aeruginosa* (Tam et al., 2005). We have recently documented for the first time, an inoculum effect of *A. baumannii* on polymyxin B *in vitro* using static time-kill experiments and PKPD modeling (Akrong et al., 2021). The IE required a 17-fold increase of the PMB concentration to reach 50% of maximal effect (EC_50_) as the initial inoculum increased from 10^5^ to 10^8^ CFU/ml.

Yet the IE of *A. baumannii* on polymyxins *in vivo* remains to be investigated. Indeed, Lin *et al.* observed a decrease in the efficacy of nebulized colistin when the initial inoculum of *A. baumannii* increased from 10^7^ to 10^8^ CFU/lung (Lin et al., 2018). But IE was only investigated on rare occasions *in vivo*, with antibiotics such as marbofloxacin (Ferran et al., 2009), piperacillin-tazobatam (Harada et al., 2014), ertapenem (Maglio et al., 2005), meropenem (Mizunaga et al., 2005) or colistin (Fantin et al., 2019) against various Gram-negative pathogens such as *E. coli*, *K. pneumoniae* or *P. aeruginosa* but never against *A. baumannii*. In these *in vivo* studies, the impact of inoculum size was evaluated based on bacterial counts at 24 or 48 h after the start of antibiotic therapy (Maglio et al., 2004; Maglio et al., 2005; Ferran et al., 2009; Lee et al., 2013; Harada et al., 2014), or on the survival of infected animals (Mizunaga et al., 2005; Fantin et al., 2019). However, to our knowledge, no PKPD modeling of the impact of inoculum size on antibiotic activity has ever been performed *in vivo*.

Therefore, our objectives were, first to evaluate whether the IE that was observed *in vitro* with *A. baumannii* and PMB could be detected *in vivo*. Second, if an *in vivo* IE was revealed, we aimed to assess the capability of the PKPD model developed with *in vitro* data (Akrong et al., 2021) to describe the newly produced *in vivo* data.

## Materials and Methods

### Chemicals and Bacterial Isolates

Polymyxin B sulfate (PMB) and cyclophosphamide monohydrate obtained from Sigma-Aldrich (Merck KGaA, Saint-Quentin Fallavier, France) were used to prepare solutions in sterile conditions. During this study, all chemicals and reagents used were analytical grade.

A clinical strain of *A. baumannii* (CS01), isolated from a patient with a meningitis (Seville, Spain) before treatment with CST, was used during this study (López-Rojas et al., 2013). Before each experiment, the strain was cultured in 5 ml of cation adjusted Muller-Hinton broth II (MHB) (Biomérieux, Marcy-l’Etoile, France) and incubated overnight at 37 ± 2°C with constant shaking (150–170 rpm). This overnight suspension was diluted 1:50 in MHB and was incubated with constant shaking at 35°C during 2 h until an OD_600nm_ of 0.26 was achieved (Ultrospec10, Biochrom Ltd., Cambridge, United Kingdom), corresponding to a bacterial count of 10^8^ CFU/ml in exponential growth phase. The bacterial suspension was centrifuged (3,000 rpm, 6 min), broth was removed and replaced by the same volume of sterile saline solution. This suspension was then diluted to obtain inocula of 10^7^ and 10^6^ CFU/ml. Samples of the inoculation solutions were serially diluted on saline, plated on Muller-Hinton agar plates (Biomérieux, Marcy-l’Etoile, France) and incubated overnight at 37°C.

### Neutropenic Mouse Thigh Infection Model

Animal experiments were carried out according to the EC Directive 2010/63/EU. They were approved by the local ethics committee (COMETHEA) and registered by the French Ministry of Higher Education and Research (approval numbers: 2019022216097190 and 2017072415099072). Five-week-old male Swiss RjOrl mice (n = 296) weighing 34 ± 2 g (mean ± standard deviation [SD]) were obtained from Janvier Labs (Saint-Berthevin, France). All animals were acclimatized in ventilated racks in a temperature-regulated environment with a 12 h light-dark cycle, with free access to food and water for a minimum of 5 days before the beginning of the experiment. Neutropenia was induced by intraperitoneal administrations of cyclophosphamide at 150 and 100 mg/kg, 4 days and 1 day prior to experimental infection, respectively (Landersdorfer et al., 2018). Thigh infection was induced by intramuscular administration of 0.1 ml of a bacterial suspension of 10^6^, 10^7^ or 10^8^ CFU/ml (corresponding to 10^5^, 10^6^ and 10^7^ CFU/thigh, respectively), into one of the posterior thigh muscles. Thus, three groups of mice were distinguished according to the inoculum injected (n = 76, 146 and 74 for 10^5^, 10^6^ and 10^7^ CFU/thigh, respectively) Each group was divided into two subgroups: treated (n = 48, 116 and 45 for 10^5^, 10^6^ and 10^7^ CFU/thigh, respectively) and control mice (n = 28, 30 and 29 for 10^5^, 10^6^ and 10^7^ CFU/thigh, respectively).

### Polymyxin B Treatment

Two hours after bacterial inoculation, mice received either a single subcutaneous administration of PMB (1, 15 or 40 mg/kg for mice infected with 10^6^ and 10^7^ CFU/thigh, and 15 or 40 mg/kg for mice infected with 10^5^ CFU/thigh), or a subcutaneous administration of saline solution (control group).

### Polymyxin B Pharmacokinetics

The PK of PMB was determined in neutropenic mice infected with the bacterial inoculum size of 10^6^ CFU/thigh (n = 56). Mice were anesthetized by isoflurane (AbbVie, Rungis, France) inhalation (3%) for 5 min at each sampling time. Blood samples were collected by intracardiac puncture into heparinized tubes up to 24 h after PMB administration for a total of 7 time points per dose level (n = 3 animals per time point). Plasma was separated from the whole blood after centrifugation at 4,000 rpm for 10 min at 4°C and divided into two samples. The first sample was used to determine total PMB concentrations and the second one (0.15 ml) was ultrafiltered (4000 rpm for 30 min at room temperature) using Centrifree^®^ ultrafiltration devices from Millipore (Merck KGaA) to determine unbound PMB concentrations and consequently protein binding. The non-specific binding of PMB to the membrane of the Centrifree^®^ ultrafiltration devices (Sader et al., 2012) was determined by ultrafiltration of PMB solutions in phosphate buffer (pH7.2) at concentrations ranging from 0.2 to 7.5 mg/L and was used to correct ultrafiltrate concentrations. Plasma samples and ultrafiltrates were stored at −20°C until further analysis. Total and unbound PMB concentrations were determined by a liquid chromatography tandem mass spectrometry (LS-MS/MS) method (Supplemental Material).

### Polymyxin B Pharmacodynamics

Mice (n = 240) were sacrificed at 6 different time points: just prior to the start of the therapy (0 h) and at 2, 4, 6, 8 and 24 h after PMB administration. A total of 3-6 mice were sacrificed at each time point. Thigh muscles were collected and homogenized with 1 ml of sterile saline solution using potters Elvehjem-type tissue grinders (Thermo Fischer Scientific, Illkirch-Graffenstaden, France). Homogenates were serially diluted in saline, plated on Muller-Hinton agar plates and incubated overnight at 37°C. Bacterial colonies were counted and expressed as log_10_ numbers of CFU/thigh. The lower limit of quantification (LOQ) was set to 800 CFU/ml corresponding to 2.9 log_10_ CFU/thigh.

### Pharmacokinetic-Pharmacodynamic Model

A PKPD model was developed in two steps to quantify the exposure-effect relationship of PMB in infected mice. First, time courses of total and unbound PMB concentrations were modeled and then, PK parameters were fixed during development of the PD part of the PKPD model.

Different structural models including one, two or three compartments, linear, nonlinear (Michaelis-Menten) or parallel linear/nonlinear elimination were evaluated to describe PK data. Models with linear and nonlinear absorption were also tested. Additive, proportional and exponential residual error models were explored.

The structural model for the bacterial population included one compartment representing drug-susceptible growing bacteria. A logistic function was used to model the self-limiting growth observed *in vivo*:

$$\frac{dB}{dt}=\;k_{net}\times \left(1-\frac{B}{Bmax}\right)\times B$$ (1)

Where, B (CFU/thigh) is the drug-susceptible bacterial population, k_net_ (h^−1^) is the apparent (net) growth rate constant and B_max_ (CFU/thigh) the maximum bacterial count reached in the tissue. The residual error was additive on a log_10_ scale for bacterial counts (log_10_ CFU/thigh).

Predicted unbound plasma concentrations were linked to the bacterial sub-model using a mathematical function to characterize PMB antimicrobial effect (k_PMB_) such as:

$$\frac{dB}{dt}=\;k_{net}\times \left(1-\frac{B}{{\text{B}}_{\text{max}}}\right)\times B\;-\;k_{PMB}\times B$$ (2)

Where multiple functions (linear, power, basic E_max_ or a sigmoidal E_max_ function) for k_PMB_ were tested.

Empirical mathematical functions (*i.e.* linear, exponential and power) describing the relationship between k_PMB_ and inoculum size were tested as a way to include the impact of inoculum size on PMB bactericidal activity.”

Model selection was based on objective function value (OFV) and goodness of fit (GOF) plots. When two models were nested, a decrease in OFV of at least 3.84 (chi square 1df *p* = 0.05) was needed to select the most complex model. Visual predictive checks (VPCs) based on 1,000 simulations were drawn after stratification on the PMB dose and the starting inoculum to evaluate the predictive performance of the model and were taken into account for model selection. Data below the LOQ were taken into account during parameter estimation by applying Beal’s M3 method (Beal, 2001). Parameter estimation was performed using NONMEM software (ICON, Dublin, Ireland) version 7.4.2 using the LAPLACIAN algorithm. Uncertainty around population parameters was estimated using the sampling importance resampling (SIR) technique (Dosne et al., 2016).

### Sensitivity Analysis

To evaluate the impact of outliers on parameter estimates and model-based inferences, a sensitivity analysis was performed. Briefly, model estimation was performed with the complete dataset and with a reduced dataset excluding outliers. Parameter estimates were compared and simulations of expected bacterial counts for all tested experimental conditions (*i.e.* all inocula and dosing regimens) were performed with the two parameter estimate sets.

## Results

### Polymyxin B Pharmacokinetics Study

A saturable non-specific binding (NSB) of PMB to the ultrafiltration membrane, translating to a non-linear decrease of PMB NSB when PMB concentration increased, was observed (Supplementary Figure S1) and could be described by the following equation:

NSB = 0.47−0.16×ln(UF) (3)

Where NSB corresponds to the non-specific binding (%) and UF to PMB concentrations in ultrafiltrates (mg/L). Equation 3 was used to correct ultrafiltrates concentrations, as follows:

$$Unbound\;concentrations\;=\;\frac{UF}{1-NSB}$$ (4)

Total and unbound PMB plasma concentrations versus time profiles in thigh-infected mice are shown on Supplementary Figure S2. Unbound concentrations in mice receiving a subcutaneous dose of 1 mg/kg were all below LOQ (unbound LOQ = 0.62 mg/L after correction by the non-specific binding). The plasma peak was smoother and delayed as the dose increased, with a time to peak (T_max_) of 0.5 h for a dose of 1 mg/kg and 2 h for a dose of 40 mg/kg. Total PMB peak concentration (C_max_) did not change proportionally with dose, but increased only 15-fold (from 0.92 ± 0.14 (mean ± SD) to 13.75 ± 0.81 mg/L) when the dose increased from 1 to 40 mg/kg, attesting for some degree of PK non-linearity across this range of PMB subcutaneous doses.

**FIGURE 1:**
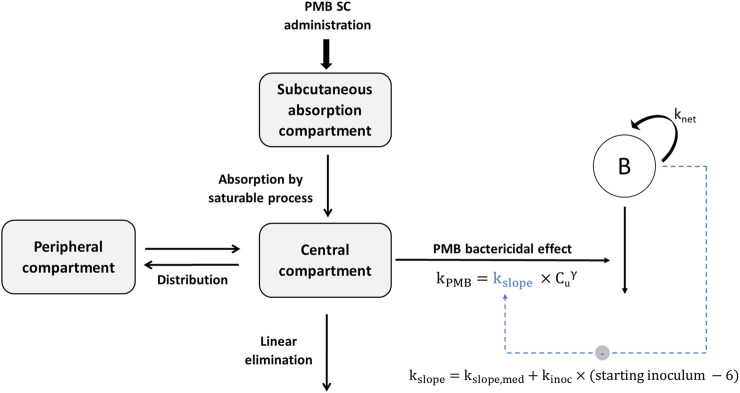
Schematic representation of the final pharmacokinetic-pharmacodynamic model characterizing the inoculum effect of *A. baumannii* on PMB bactericidal activity (k_PMB_). SC, subcutaneous; k_net_, apparent growth rate constant of bacteria; k_slope_, kill rate constant due to PMB; k_slope,med_, kill rate constant for a theoretical starting inoculum of 6 log_10_ CFU/thigh; k_inoc_, constant describing the inoculum effect on k_slope_; Cu, unbound PMB concentration; γ, power parameter for PMB effect.

**FIGURE 2:**
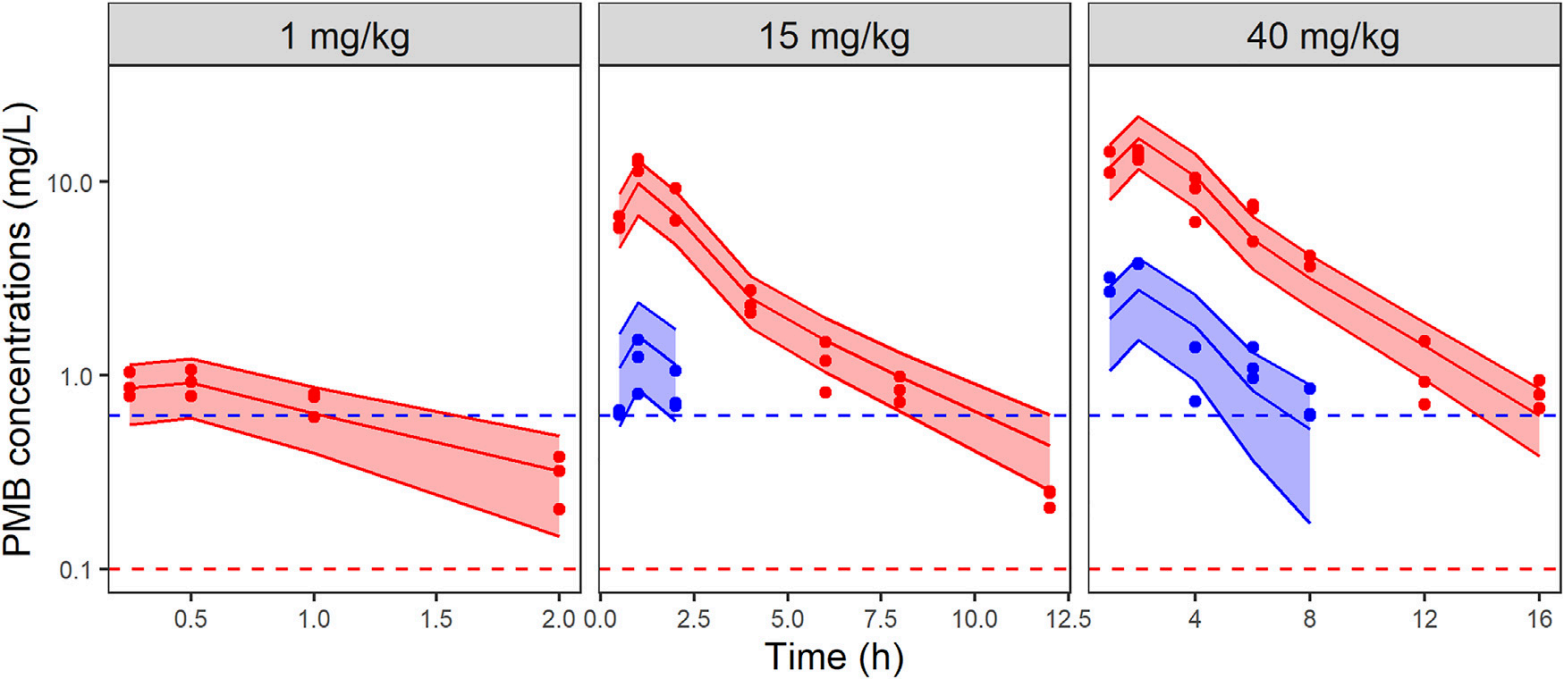
VPCs of the final PK model for total (red) and unbound (blue) PMB plasma concentrations, stratified by dose. Circles represent observed data, solid lines represent the median of the simulations and the colored-shaded areas depict the 80% prediction intervals for 1,000 simulated profiles. Dashed lines correspond to the limits of quantification (0.1 mg/L and 0.62 mg/L for total (red) and unbound (blue) concentrations, respectively). Note the different axis scales.

Total and unbound plasma PMB concentrations versus time were best fitted by a two-compartment model with saturable absorption from the injection site, and linear elimination (Figure 1). Parameter estimates with their corresponding uncertainties are summarized in Table 1. PMB plasma protein binding was concentration independent within the observed range of total concentrations (0.20–14.56 mg/L) and the unbound fraction, estimated to be 17% (Table 1), was used for unbound concentrations fitting. GOF plots (Supplementary Figure S3) and VPCs (Figure 2) demonstrate that the selected model adequately predicted the mean tendency and dispersion of the total plasma data across the investigated dose range. For unbound concentrations, the model slightly overestimates and underestimates peak concentrations after the 15 and 40 mg/kg doses respectively (Supplementary Figure S3).

**TABLE 1:** Parameter estimates and relative standard errors for the final PK model.

Parameter	Unit	Estimate (%RSE)
Maximum absorption rate	mg/h/kg	14.7 (10.3)
Amount in the subcutaneous compartment that produces 50% of the maximum absorption rate	mg/kg	2.24 (41.4)
Clearance	L/h/kg	0.437 (3.9)
Distribution volume of the central compartment	L/kg	0.740 (16.7)
Distribution volume of the peripheral compartment	L/kg	0.743 (16.4)
Intercompartmental clearance	L/h/kg	0.315 (27.3)
Fraction unbound	-	0.166 (9.3)
Proportional residual error for total concentrations	%	24 (11.4)
Additive residual error for total concentrations	mg/L	0.0115 (40.6)
Proportional residual error for unbound concentrations	%	35 (14.7)
Additive residual error for unbound concentrations	mg/L	0.0457 (36.8)

RSE, Relative Standard Error

### Polymyxin B Pharmacodynamic Study

**FIGURE 3:**
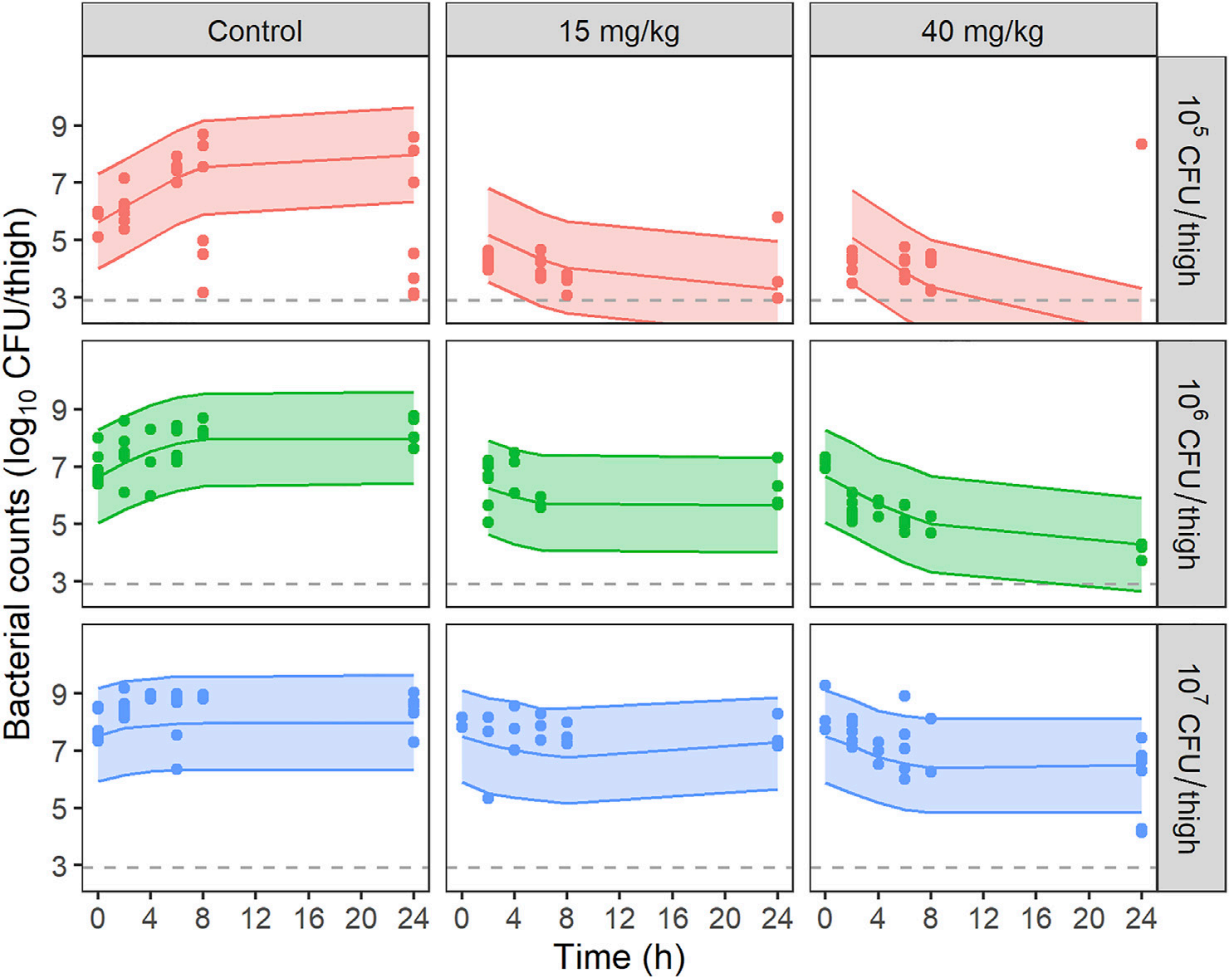
VPCs of the final PKPD model for bacterial counts, stratified by dose of PMB and starting inoculum. Circles represent experimental data, solid lines the median of simulated data and, colored areas depict the 80% prediction intervals for 1,000 simulated profiles. Dashed lines correspond to the limit of quantification (2.9 log10 CFU/thigh).

All untreated infected animals in the control group survived after 24 h. The time courses of bacterial loads after a single dose of PMB at 15 and 40 mg/kg are shown in Figure 3 for the three inocula. At the start of PMB treatment (2 h post-infection), bacterial counts were equal to 5.8 ± 0.4, 7.1 ± 0.5 and 8.0 ± 0.5 log_10_ CFU/thigh in mice infected with 1.5 × 10^5^, 1.4 × 10^6^ and 1.3 × 10^7^ CFU/thigh inoculum, respectively. For the 10^5^ CFU/thigh inoculum, bacterial counts in the untreated control group increased for the first 6 h for all animals but two patterns are seen at 8 and 24 h post-infection. For half of the studied mice bacteria reached a plateau at 8 h (8.2 ± 0.6 log_10_ CFU/thigh). For the other half, unexpectedly low bacterial counts were observed (8 h: 4.2 ± 0.9 log_10_ CFU/thigh and 24 h: 3.6 ± 0.7 log_10_ CFU/thigh). At higher inocula, bacterial counts in the untreated control group increased until a plateau was reached 8 h (8.3 ± 0.3 log_10_ CFU/thigh) and 4 h (8.9 ± 0.1 log_10_ CFU/thigh) after infection with the 10^6^ and 10^7^ CFU/thigh inoculum, respectively. When infected mice were treated with PMB at 1 mg/kg, no differences in bacterial counts with the control group were observed (data not shown). For the 10^5^ CFU/thigh inocula high bacterial load reductions of 2.6 ± 1.2 log_10_ CFU/thigh were observed 24 h after administration of 15 mg/kg PMB while a moderate efficacy of PMB was observed at 15 mg/kg for the two highest inocula (Figure 3). In contrast high reductions of 2.5 ± 2.3, 3.5 ± 0.9 and 2.1 ± 1.4 log_10_ CFU/thigh were observed 24 h after administration of 40 mg/kg PMB for 10^5^, 10^6^ and 10^7^ CFU/thigh inocula respectively.

### Polymyxin B PK-PD Study

The time course of bacterial counts was adequately described by the model depicted on Figure 1. Parameter estimates with their corresponding uncertainties are summarized in Table 2. VPCs of the final model are shown on Figure 3 and GOF plots on Supplementary Figure S4. PMB bactericidal effect (k_PMB_) was best described by a power function:

$$k_{PMB}\;=\;k_{slope}\;\times \;{\text{C}}_{\text{u}}^{\text{γ}}$$ (5)

Where k_slope_ corresponds to the kill rate constant due to PMB (L/mg.h), C_u_ the unbound PMB concentration (mg/L) and  γ , the power parameter for PMB effect.

**TABLE 2:** Parameter estimates and relative standard errors (RSE) for the final PMB PKPD model.

Parameter	Unit	Estimate (%RSE)
k_net_: Apparent growth rate constant	h^−1^	0.594 (16.6)
B_max_: Maximum bacterial count reached in the tissue	Log_10_ CFU/thigh	8.00 (2.0)
k_slope,med_: Kill rate constant due to PMB for a theoretical median starting inoculum of 6.5 log_10_ CFU/thigh	L/mg.h	1.00 (12.1)
γ: Power parameter for PMB effect	-	0.162 (20.1)
k_inoc_: Constant describing the inoculum effect on k_slope,med_	-	−0.194 (22.3)
σ: Additive residual error on the log_10_ scale for total bacterial count	Log_10_ CFU/thigh	1.63 (9.4)

The inoculum effect was incorporated in the model as a decrease of k_slope_ with increasing theoretical starting inoculum using a linear function:

$$k_{slope}=k_{slope,med}\;+k_{inoc}\times \left(starting\;inoculum-6\right)$$ (6)

Where k_slope,med_ is the kill rate constant for a theoretical starting inoculum of 6 log_10_ CFU/thigh corresponding to the median of the starting inoculum tested in the present study (5, 6 or 7 log_10_ CFU/thigh) and k_inoc_ is the constant describing the inoculum effect on k_slope_.

A decrease of k_slope_ from 1.19 to 0.81 L/mg.h (−32%) was predicted for a starting inoculum increasing from 5 to 7 log_10_ CFU/thigh respectively. k_PMB_ at various PMB concentrations was derived from Equation 5, using for each starting inoculum the corresponding k_slope_ value, and converted into initial killing half-lives (IK-HL) (Table 3), as previously performed with the *in vitro* model (Akrong et al., 2021).

**TABLE 3:** Model derived initial killing half-lives (min) at various unbound PMB concentrations and starting inocula.

	PMB concentration (mg/L)				
Inoculum (CFU/thigh)	0.5	1	2	3	4
10^5^	39	35	31	29	28
10^6^	47	42	37	35	33
10^7^	58	52	46	43	41

### Sensitivity Analysis

The data-points identified as outliers were samples with less than 10^5^ CFU/thigh from the control group infected with inoculum 10^5^ CFU/thigh at times 8 and 24 h. With the sensitivity analysis results, it was observed that these outliers were not influential regarding parameter estimates and simulations under the final model. Detailed results can be found in supplemental material (Supplementary Table S1 and Supplementary Figure S5).

## Discussion

These new study results can be compared with those previously obtained in relatively similar conditions, at least from PK standpoint (Landersdorfer et al., 2018). In both studies PMB was administered subcutaneously to neutropenic mice infected with *K. pneumoniae* (Landersdorfer et al., 2018) or *A. baumannii*, leading to saturable absorption rate. However although Landersdorfer *et al.* used a model with parallel linear and saturable absorption, an Emax model was sufficient to provide satisfactory fit of our data. Noticeably, while this nonlinear absorption was important to consider for PK and then PKPD modeling of these animal data, it would not be relevant in clinical practice since PMB is administered intravenously and not subcutaneously. Another difference was observed between these two studies in terms of elimination. We observed linear elimination characterized by a clearance value, whereas Landersdorfer *et al.* described their data, with again a model including parallel linear and saturable pathways. Doses ranging were comparable between these two studies (from 2 to 32 mg/kg for Landersdorfer *et al.* and from 1 to 40 mg/kg for us), and peak concentrations observed in Landersdorfer *et al* study at the highest dose, were close to 20 mg/L and therefore only slightly higher than in our study (15 mg/L).

Plasma protein binding results also demonstrate some discrepancies between the two studies. Although both studies showed extensive binding, close to 80 and 90%, our estimated unbound fraction (0.17 on average) was twice the previously reported value at 0.086 (Landersdorfer et al., 2018). This discrepancy may be explained by differences in methodology. We used ultra-filtration after correction for non-specific adsorption on membranes to determine unbound fraction individually in infected mice, whereas the previous study used ultracentrifugation and pooled plasma sampled drawn from infected mice and spiked with PMB. The latter methodology may seem more suitable for the determination of protein binding of drugs, such as PMB, that exhibit significant nonspecific binding to laboratory material including ultrafiltration membranes (Cheah et al., 2015). Although, ultrafiltration has also been used to determine unbound concentrations of daptomycin, another antibiotic known to adhere to ultrafiltration membranes, after evaluation of non-specific binding by regression methods (Kim et al., 2008; Grégoire et al., 2019). This two-fold difference in unbound fractions should complicate PKPD modeling comparisons between these two studies, but not the IE investigated during this new study.

**FIGURE 4:**
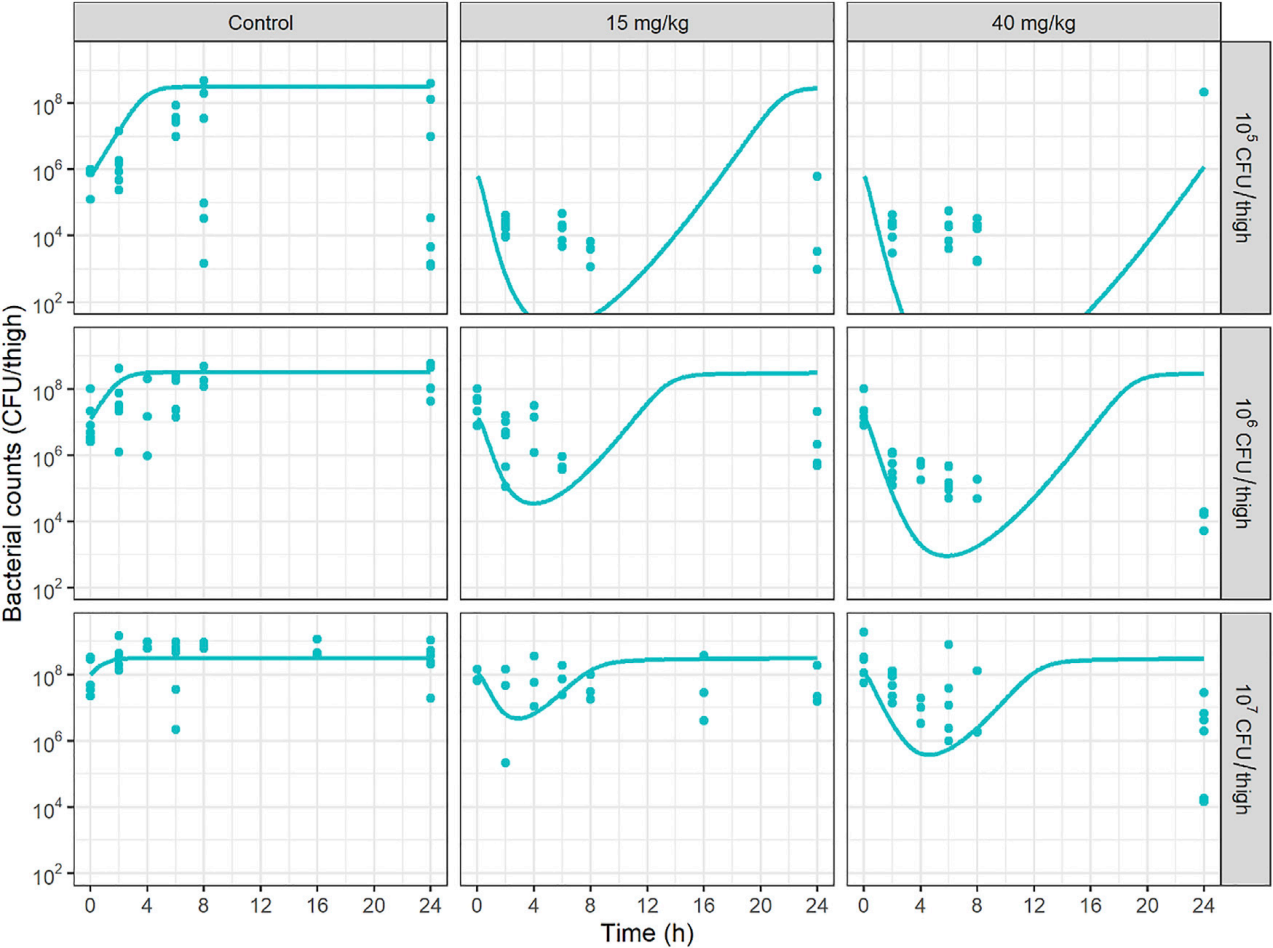
Bacterial counts (CFU/thigh) versus time profiles at various initial inocula predicted by combining the PMB mice PK model, with the PD model previously developed to characterize the inoculum effect *in vitro* (Akrong et al., 2021). Solid circles represent experimental data and solid lines represent model predictions.

PKPD results of this new study could not be compared with Landersdorfer *et al.*, not only because the bacterial species were different, but also because we have performed repeated measurements of bacterial counts over time, to describe a bacterial count versus time profile, which constitutes a major originality of our study. We have first compared the bacterial count versus time profiles at various initial inocula, with those predicted by combining our PK model in mice previously discussed, with the PD model that we previously developed to characterize the IE *in vitro* (Akrong et al., 2021). Such promising simulations can be found in publications that apply modelling to *in vitro* time-kill and/or hollow-fiber data (Yadav et al., 2015; Ly et al., 2016; Mohamed et al., 2016; Kristoffersson et al., 2019). However *in vivo* data are missing to evaluate the predictive ability of those simulations. In the present study, *in vivo* data show (Figure 4) that initial CFU decay with time is less rapid than predicted by the *in vitro* PD model, but more importantly the rapid regrowth observed *in vitro* was no more apparent *in vivo*. These *in vitro*—*in vivo* discrepancies invite caution when making recommendations based on predictions of models based solely on *in vitro* data. The reasons for these discrepancies should be further investigated and the importance of performing *in vivo* experiments is reinforced. These differences in model are schematically represented on Supplementary Figure S6.

An innovative aspect of this study was the refinement of a PKPD model, based on the model developed after *in vitro* TK experiments conducted with *A. baumannii* and PMB (Akrong et al., 2021). The comparison between PD parameter values obtained after *in vitro* and *in vivo* data fitting indicated that the apparent growth rate constant was lower *in vivo* (0.594 h^−1^) (Table 2) than *in vitro* (1.62 h^−1^) (Akrong et al., 2021), as previously shown for *E. coli* (0.76 versus 1.30 h^−1^) (de Araujo et al., 2011). The different growth behavior between *in vitro* and *in vivo* has been associated in literature to the different environmental conditions, with higher volumes and nutritional factors more abundantly available *in vitro* than *in vivo* leading to a medium favorable to bacterial growth (Gloede et al., 2010; Mouton, 2018). As an illustration, the bactericidal effect of PMB characterized by the typical killing rates (k_PMB_), described by a power function *in vivo* and by a sigmoid Emax model *in vitro*, were respectively equal to 1.25 h^−1^ and 6.55 h^−1^ for initial inocula of 10^6^ CFU/thigh and 10^6^ CFU/ml and PMB concentration of 4 mg/L, that corresponds almost to the unbound peak concentration of PMB after a dose of 40 mg/kg. Similarly, a two times smaller maximum killing effect was previously observed for piperacillin against *E. coli* in a murine thigh infection model as compared to *in vitro* (de Araujo et al., 2011).

In both *in vitro* and *in vivo* PKPD models, a decrease in PMB killing effect was related to the baseline inoculum and was modeled either as a decrease of the *in vivo* PMB killing rate constant (k_slope_) or as an increase of the *in vitro* half-maximal effective concentration of PMB (EC_50_), making PMB IE difficult to compare between *in vitro* and *in vivo*. *In vivo* IK-HL were derived from Equation 5 for each starting inoculum and various PMB concentrations to better illustrate the consequences of IE on PMB activity (Table 3). In the present study, the modelling suggests that the *in vivo* IE is moderate and not concentration dependent with a mean IK-HL 48% higher at 10^7^ CFU/thigh compared with 10^5^ CFU/thigh inoculum (48 vs. 32.4 min). In contrast, our *in vitro* model suggested that the IE was PMB-concentration dependent and attenuated at high PMB concentrations (Akrong et al., 2021). As an example, at a PMB concentration equal to 4 mg/L, *in vitro* IK-HL increased by 60% (from 5 to 8 min) when the starting inoculum increased from 10^5^ to 10^7^ CFU/ml, whereas it increased by 140% (from 10 to 24 min) for a PMB concentration 16 times lower (0.25 mg/L).

The clinical relevance of *in vitro* IE has been questioned in previous studies (Maglio et al., 2004; Maglio et al., 2005; Fantin et al., 2019). Indeed, in the case of cefepime (Maglio et al., 2004) and ertapenem (Maglio et al., 2005) *in vitro* elevations of *E. coli* MICs were seen with an initial inoculum at 10^7^ CFU/ml compared to 10^5^ CFU/ml, while no IE was observed *in vivo* (neutropenic mouse thigh infection model). On the other hand, Fantin *et al.* observed an *in vivo* IE in mice infected with *E. coli* and treated by colistin (Fantin et al., 2019).

In conclusion, a PKPD model has been successfully developed to characterize the *in vivo* IE of *A. baumannii* on PMB, which confirms the IE observed *in vitro*. The PKPD model previously developed from *in vitro* TKC data was modified to take into account the intrinsic differences between *in vitro* and *in vivo* experimental infection models. The comparison between *in vitro* and *in vivo* PKPD parameters was not straightforward, especially due to the absence of *in vivo* regrowth. Yet although less pronounced than *in vitro*, the initial inoculum size of *A. baumannii* had a real impact on *in vivo* PMB activity.

## Data Availability

The raw data supporting the conclusions of this article will be made available by the authors, without undue reservation.
