# Supplementary material for: PKPD Modeling of the Inoculum Effect of Acinetobacter baumannii on Polymyxin B in vivo
Source: Front Pharmacol. 2022 Mar 16;13:842921. doi: 10.3389/fphar.2022.842921 (PMC8966651; doi:10.3389/fphar.2022.842921)
Supplement: Supplementary file 1 [file DataSheet1.docx]

**SUPPLEMENTAL MATERIAL**

**MATERIEL AND METHOD**

**LC-MS/MS method**

Briefly, 0.1 mL of plasma or 0.05 mL of ultrafiltrates added to 0.05 mL of drug-free plasma, were mixed with 0.75 mL of phosphate buffer (pH 7.2) and 0.01 mL of internal standard (colistin sulfate ; Merck KGaA) at 6.25 mg/L for plasma and 5 mg/L for ultrafiltrates. The samples were briefly vortexed and then centrifuged at 3000 rpm for 5 min. The supernatants (0.800 mL) were loaded onto SPE columns (Oasis HLB solid-phase extraction (SPE) cartridges (1 mL, 30 mg), Waters, Saint-Quentin-en-Yvelines, France), preconditioned with 1 mL of methanol followed by 1 mL of water. Then the columns were washed with 1 mL of water and dried under a nitrogen pressure. The analytes were eluted with 0.5% formic acid in methanol. They were evaporated at 45°C under a gentle nitrogen jet stream. The residues were dissolved in 0.1 mL of 0.1% formic acid in water and analyzed by LC-MS/MS. The system included an Alliance Waters 2695 liquid chromatography system module (Waters) coupled with an API Quattro Micro (Waters). PMB was analyzed on an XBridge C_18_ column (5 µm, 2.1 x 150 mm; Waters). The mobile phase A consisted of 0.1% formic acid in water, and mobile phase B was 0.1% formic acid in acetonitrile. The gradient for mobile phase A and B were respectively set at 75 and 25% with a flow rate of 0.2 mL/min. Electrospray ionization in positive mode was used for the detection of PMB. Ions were analyzed in the multiple reaction monitoring, and the following transitions were inspected: *m/z* 602.1→101 for PMB1, *m/z* 595.1→101 for PMB2, and *m/z* 585.1→101 for CSTA and *m/z* 578.1→101 CSTB. Calibration curves of PMB ranged from 0.1 to 10 mg/L for plasma and ultrafiltrates, and were quantified with a quadratic regression mode. The intraday variability was characterized at four levels (0.3, 1, 2.5 and 10 mg/L) with a precision and bias of <20% for the lowest level, and < 15% for the others.

**RESULTS**

**
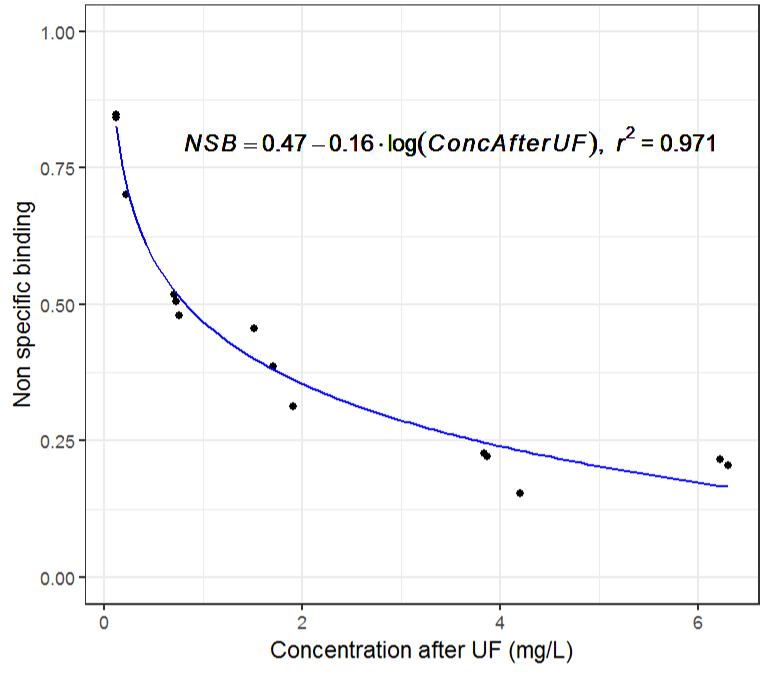
**

Figure S1. Non-specific binding of PMB versus PMB concentration after ultrafiltration.


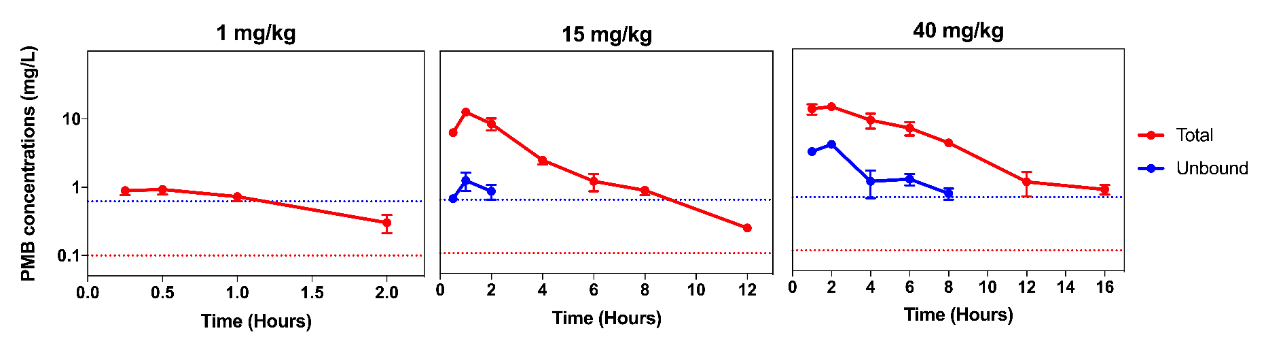


Figure S2. Total (red) and unbound (blue) plasma PMB concentrations versus time after the administration of a single subcutaneous dose of 1, 15 or 40 mg/kg in mice infected with the lower inoculum (10^6^ CFU/thigh). Data points represent mean ± SD (n=3 per time point). Dashed lines correspond to the limits of quantification (0.1 mg/L and 0.62 mg/L for total (red) and unbound (blue) concentrations, respectively). Note the different x-axis scales.


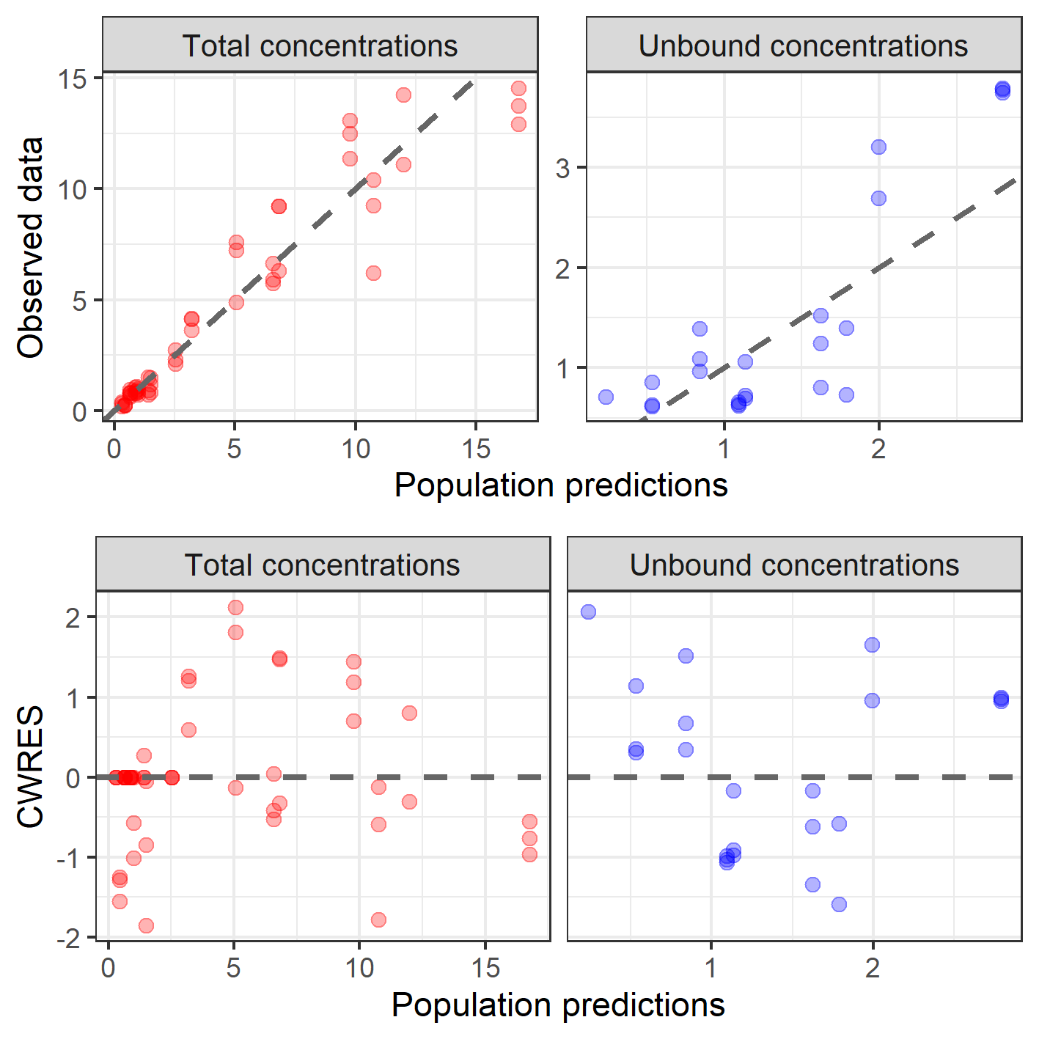


Figure S3. Goodness of fit plots (GOF) of the final PK model for total (red) and unbound (blue) concentrations. A and B: observed data versus population predicted concentrations. C and D: conditional weighted residuals (CWRES) versus population predictions.


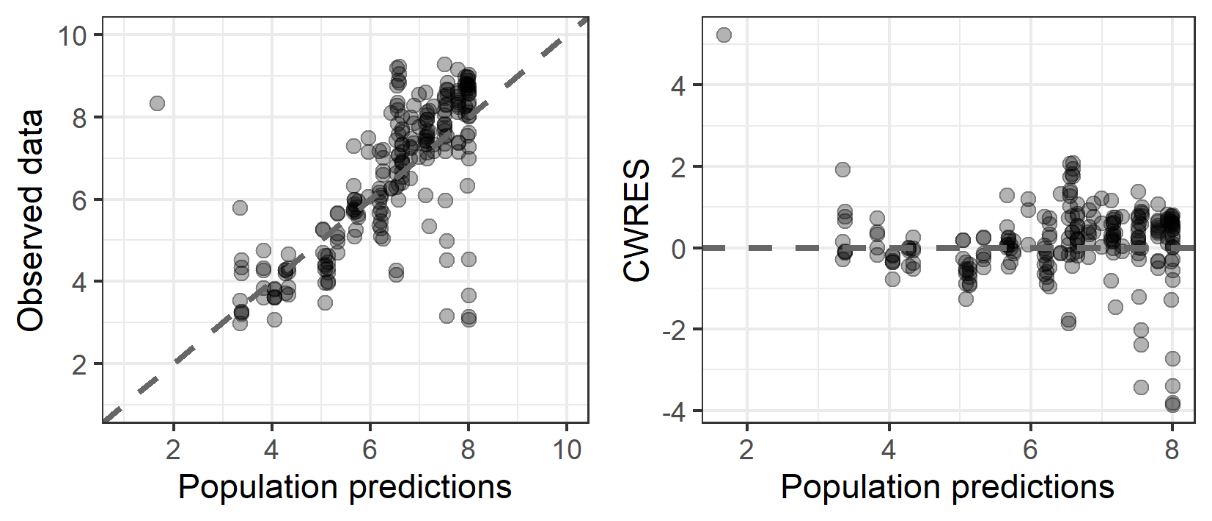


Figure S4. Goodness of fit plots (GOF) of the final PKPD model. A: observed data versus population predicted bacterial counts. B: conditional weighted residuals (CWRES) versus population predictions.


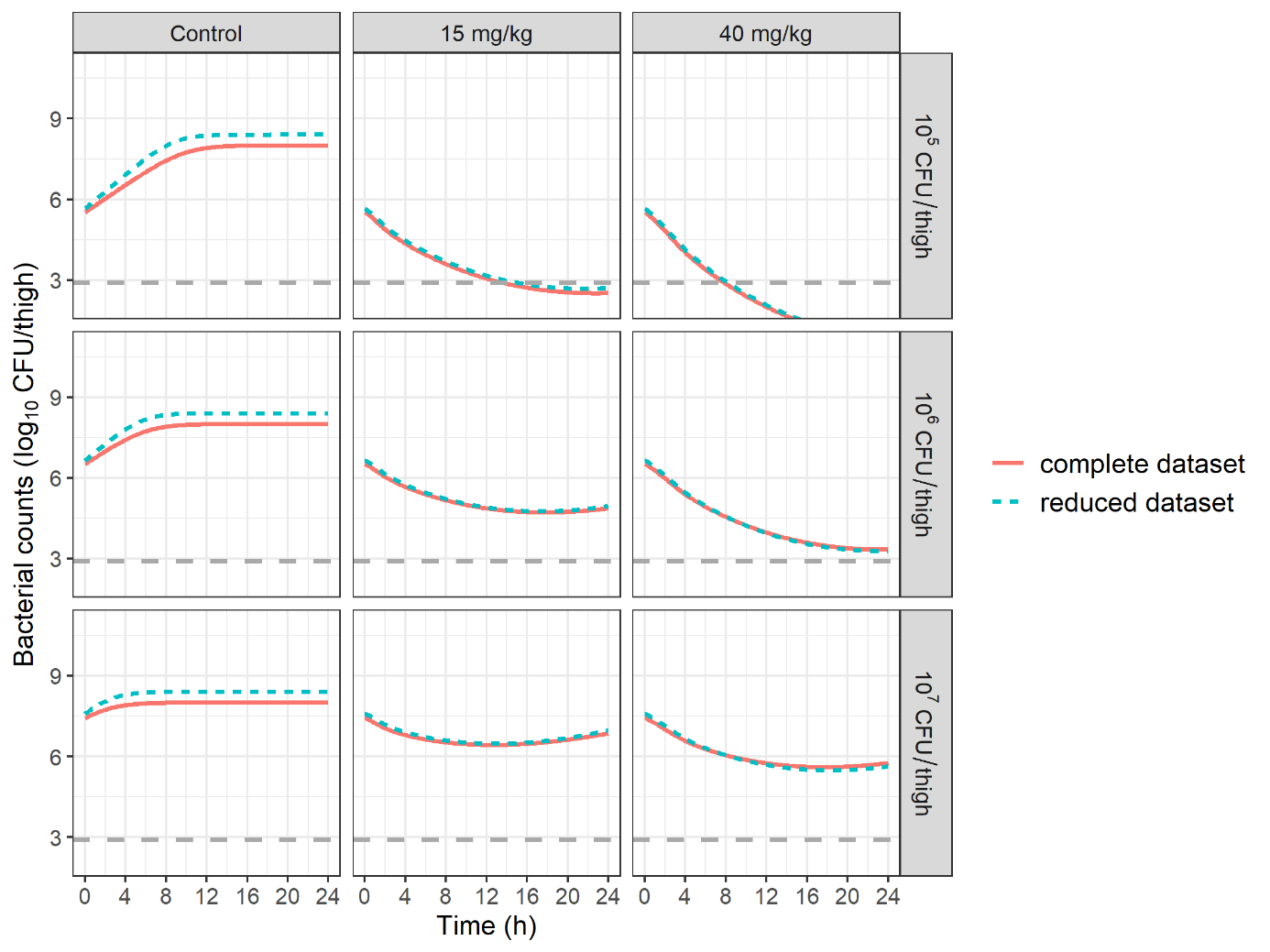


Figure S5. Simulation of expected bacterial counts under the final model using parameters estimated with the complete dataset (full red line) and parameters estimated with the reduced (without outliers) dataset (dashed blue line). The grey dashed line represents the limit of quantification (800 CFU/thigh).


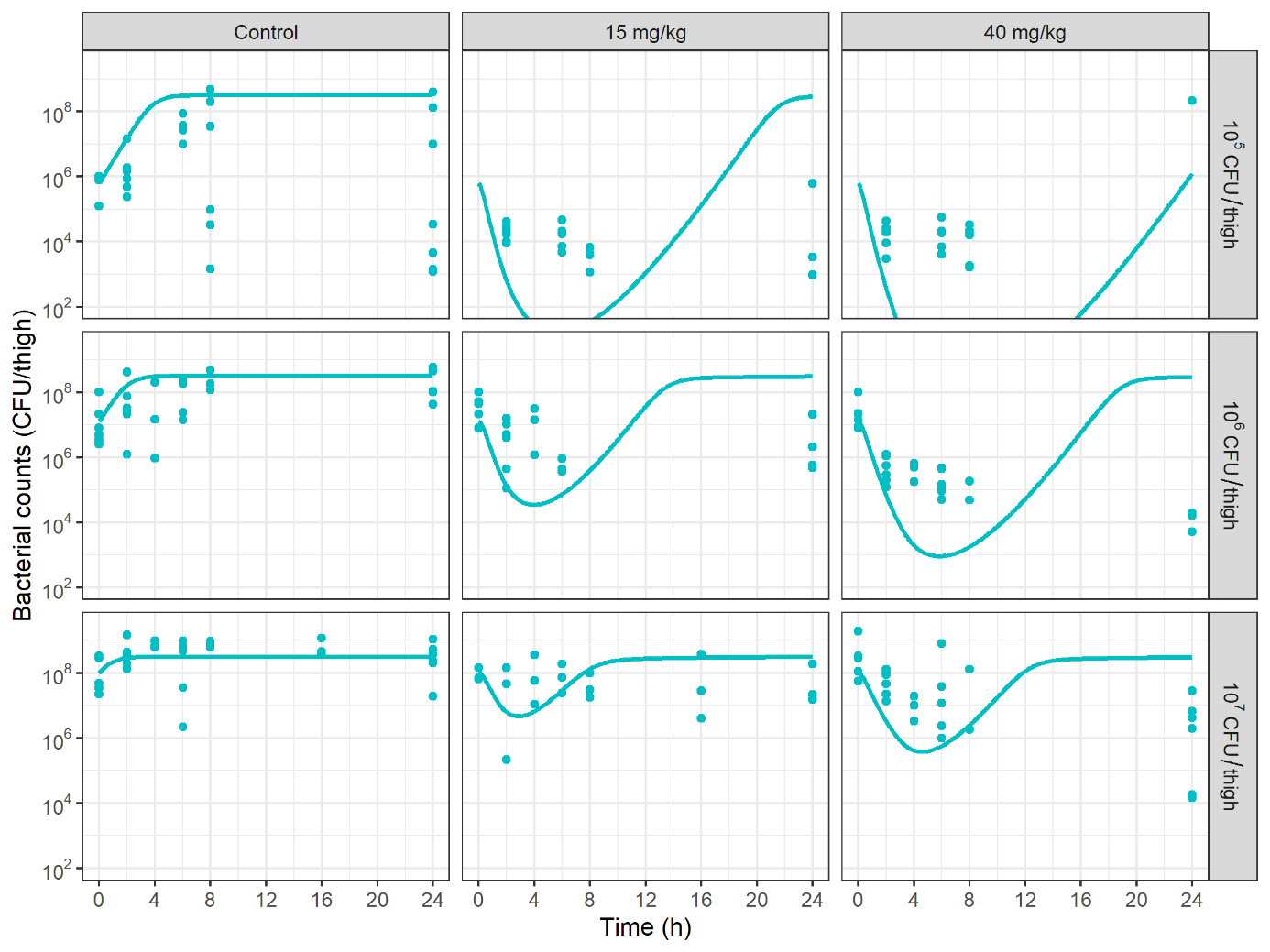


Figure S6. Bacterial counts (CFU/thigh) versus time profiles at various initial inocula predicted by combining the PMB mice PK model, with the PD model previously developed to characterize the inoculum effect *in vitro* (Akrong et al., 2021)*.* Solid circles represent experimental data and solid lines represent model predictions.

| Table S1. Comparison of parameter estimates with the complete dataset and a reduced dataset excluding outliers | | | |
| --- | --- | --- | --- |
|  |  | **Estimate (%RSE)** | |
| **Parameter** | **Unit** | Complete dataset | Reduced dataset |
| k_net_: Apparent growth rate constant | h^-1^ | 0.594 (16.6) | 0.74 (10.2) |
| B_max_: Maximum bacterial count reached in the tissue | log_10_ CFU/thigh | 8.00 (2.0) | 8.4 (1.7) |
| k_slope,med_: Kill rate constant due to PMB for a theoretical median starting inoculum of 6.5 log_10_ CFU/thigh | L/mg.h | 1.00 (12.1) | 1.19 (8.0) |
| γ: Power parameter for PMB effect | - | 0.162 (20.1) | 0.147 (13.1) |
| k_inoc_: Constant describing the inoculum effect on k_slope,med_ | - | -0.194 (22.3) | -0.172 (17.2) |
| σ: Additive residual error on the log_10_ scale for total bacterial count | log_10_ CFU/thigh | 1.63 (9.4) | 1.1 (9.8) |

**REFERENCES**

Akrong, G., Chauzy, A., Aranzana-Climent, V., Lacroix, M., Deroche, L., Prouvensier, L., et al. (2021). A new PKPD model to characterize the inoculum effect of Acinetobacter baumannii on polymyxin B in vitro. *Antimicrobial Agents and Chemotherapy*. doi:10.1128/AAC.01789-21.
